# Competition between social and market renting: a theoretical application of the structure-conduct-performance paradigm

**DOI:** 10.1007/s10901-012-9276-7

**Published:** 2012-03-17

**Authors:** Christian Lennartz, Marietta Haffner, Michael Oxley

**Affiliations:** 1 OTB Research Institute for Housing, Urban and Mobility Studies, Delft University of Technology, Jaffalaan 9, Delft, 2628 BX South Holland The Netherlands; 2 Centre for Comparative Housing Research, De Montfort University, The Gateway, Leicester, LE1 9BH UK

**Keywords:** Competition, Structure-conduct-performance paradigm, Rental housing markets, Social housing, Private renting

## Abstract

Housing policies in many countries have become more market orientated as the role of governments has shifted from the direct supply and funding of non-market housing towards the role of a regulator and facilitator. Central to this development is the notion that providers of social housing have to become more competitive. Arguably, these social housing changes have important implications for the relationship between social and market rented housing and thus the rental market as a whole. Conceptual frameworks that facilitate the understanding of this relationship are sparse commodities. This paper seeks to develop a theoretical framework that can be used to shed light on the conditions, processes, and effects of the new relation between the two rental tenures from an economic competition viewpoint. Therefore, this paper adapts the structure-conduct-performance paradigm to rented housing and discusses the framework’s applicability and value on a theoretical level.

## Introduction

The simultaneous developments of changing supply structures and the commercialization of social housing^1^ providers across many Western countries (Maclennan and More 1997; Whitehead 2003; Whitehead and Scanlon 2007; Scanlon and Whitehead 2008; Haffner et al. 2009) and the increasing involvement of profit-oriented landlords in the provision of rental housing for low-income households (Hulse and Pawson 2010; Retsinas and Belsky 2008; O’Sullivan and De Decker 2007) have arguably led to a convergence of the activities of social and private landlords. Housing researchers have pointed out that the blurring of landlords’ activities as well the idea that both sectors increasingly serve similar tenant groups have broadened the scope for competition between social and market rental housing^2^ (see also Hulse et al. 2010; Haffner et al. 2009). Since competition is often seen as a means to an end by policy-makers and as the effects of such a competitive relationship between rental tenures for the wider housing system have been widely ignored, there seems to be much confusion and a lack of understanding of this contemporary phenomenon. This paper seeks to add to the housing literature on the relation between (rental) housing tenures by devising an innovative conceptual framework that can be used as a guiding tool to unfold the complex relationship between social and market rental housing from a competition perspective.

Grounded in the extrapolation of various competition theories in the economic literature this paper thus presents a holistic approach of how to assess (1) the competitiveness of rental market structures, (2) the instruments and goals of housing policy with regard to competition policy in housing, (3) the competitive behaviour of landlords and tenants, (4) the system outcomes of the rental market, and (5) the empirical links between these aspects. As such, the paper is mainly at an abstract theoretical level; yet it makes some references to rental housing in practice and how it can be interpreted within the logic of the framework.

The structure of the paper is as follows. First, the paper gives a more precise account of the idea of competition between social and market rental housing in theory and practice, and how their relation has been contextualized by housing researchers in the past. Section 3 presents the logic and functionality, but also the shortcomings of the structure-conduct-performance paradigm (SCP) as the mainstream theoretical approach to the analysis of competition in commercial markets. Based on the arguments on the specifics of competition in rental housing as well as the discussion on the usefulness of the SCP framework, Sect. 4 devises the SCP of rental housing as the conceptual model for competition analysis in rental housing markets. Hereafter, the applicability of the framework is shown by delineating some market structural and behavioural aspects of inter-tenurial competition in the Netherlands, which results in the presentation of a wider research agenda. The paper concludes in Sect. 6.

## Competition and rental housing

Before answering the question of whether competition between the two rental tenures is possible at all, it seems worthwhile to take a broader look at the notion of competition on rental housing markets. First, one might ask: Competition for what? This question relates to the specific characteristic of the good housing in that it has both investment and consumption attributes. The rental dwelling can be considered as an investment good, which produces income for landlords; yet, it can also be seen as a housing service for tenants, which takes into account the physical quality of the dwelling, any locational condition and the legal quality of the rental housing consumption (Barr 1998). Breaking up the product rented housing into these two categories means that before the actual provision of a housing service takes place, landlords might compete for rental housing assets, building sites, government contracts or even resources for the construction of new housing.

The second question then is: Competition between whom? In principle, competition between the providers in rental housing markets could take place within the social housing industry, within the market rental sector, and/or between social and market rental providers. Indeed, the aim of introducing more market-oriented policies in Western countries was primarily to introduce competitive elements into the social housing industry (Walker 2000; Scanlon and Whitehead 2008).

The focus of this paper is on competition between social and private landlords to provide accommodation for tenants. This is differentiated from competition for assets and competition within each sector; however, it is acknowledged that such competition can exist and might interact with the type of competition considered here.

### A strictly theoretical perspective on competition between social and market renting

If one follows a very narrow definition of social and market rental housing, one in which the two sectors are defined by their respective allocation and adjustment mechanisms—i.e. the way suppliers and consumers come together—competition between both rental tenures appears impossible.

Under such a definition market rented housing is allocated by supply, demand, and the rent. The latter has the function of signalling shortages or oversupplies of rental services. Theoretically, rents can be adjusted to a market clearing price (Oxley 2000). A large number of market landlords operate on a profit-maximizing premise. Consumers of commercial housing services are seen as utility-maximizers. Their demand is governed by the willingness and ability to pay for a certain housing service (O’Sullivan and Gibb 2003).

Social housing, on the other hand, is primarily not allocated by financial considerations of market actors (Oxley 2000). Supply is not based on profit-maximizing decisions as in private rental markets; rather, non-pecuniary goals prevail as social housing providers are to a large extent driven by their public tasks (Whitehead and Scanlon 2007). Concurrently, demand for social housing services is not steered by tenants’ ability or willingness to pay the rent, but by a politically and socially defined and interpreted form of need (Maclennan and More 1997). Furthermore, since social housing rents are prescribed by governments and tend to be kept below market levels, they clearly have a different purpose than the signalling function of market rents. This implies that adjustment in social housing to new market circumstances remains publicly controlled and thus reflects political objectives rather than decisions of independent providers and consumers of housing services (Whitehead 2003).

When accepting these theoretical arguments, price competition between providers of social and market housing services would be impossible, since the provision of market renting starts where the provision of social housing ends, i.e. the last household not being able to afford the rent of the cheapest market dwelling. Arguably, the objectives and rationales of the two rental tenures are different in most countries. Nonetheless, the definitions of social and market housing are, of course, highly theoretical and provide only narrow descriptions of how the social and market rental industries operate.

### Housing market realities: differing forms and degrees of competition

A first argument against accepting this solely theoretical view is the way governments intervene in the private rental market through regulation policies. Housing economists (e.g. Maclennan 1982; Quigley 2003; O’Sullivan and Gibb 2003) note that landlords tend to have better knowledge of the quality of their dwellings than tenants. Adding that rental housing services are highly heterogeneous, preferences of tenants are idiosyncratic, and the transaction costs are extremely high when moving to a new dwelling, it might be assumed that competitive forces are inherently constrained on rental markets, be it within the two sectors or between them. However, these market imperfections have led to extensive regulation and operating rules with regard to rents, quality, and property rights of tenants in many countries (Arnott 1995). For instance, in Germany rent increases are regulated by the system of local reference rents (Haffner et al. 2009), while in Sweden rent setting in the private sector is circumscribed by social housing rents (Turner 2007). In the Netherlands rents for all dwellings (social and market) below the so-called liberalization threshold are subject to the same regulation system (Haffner and Boelhouwer 2006). In almost every country, including the liberal housing systems where private-sector rents are almost wholly deregulated (e.g. the UK, the United States and Australia; Hoekstra 2009; Pawson 2006), tenants are supported in their rent payments through various forms of housing allowance and new rental housing provision tends to be organized by planning authorities (for the United States see Retsinas and Belsky 2008). It follows that market rented housing is not intrinsically based on free market choices; rather it can mimic social housing markets in the way the relative power positions of tenants and landlords are controlled by public authorities.

Additionally, it is not helpful to consider market renting as one coherent sector. Market renting can have quite a fractured structure of provision, since in reality it tends to consist of different groups of landlords—divided by, inter alia, organization structures and financial goals. In this context, Rugg and Rhodes (2008, p. 15) rightly note that market renting is characterized by the existence of distinctive submarkets, in which “tenants tend to carry certain expectations, and landlords will frame their management practices and purchase property types to fit the needs of their target tenant group. These submarkets may be spatially concentrated or widely dispersed, depending on the demand group and on the supply of particular property types in a given area”. From this it follows that market renting can have different purposes within and between various countries. For instance, private renting in the UK seems to bear a certain connotation, such as a temporary first step towards owner-occupation on the housing ladder, whereas the market rental sector in Germany’s housing system conveys a tenure-for-life idea (Kemp and Kofner 2010). Nonetheless, even these two extremes share the paradigm that market rental accommodation is inhabited by all kinds of household.

This directly relates to a more recent phenomenon in market renting. Governments in many countries, such as Ireland, the UK, Belgium, and Germany, have sought to increasingly involve market rental landlords in the provision of housing for low-income households. Studying private renting in Northern Ireland, Gray and McAnulty (2008) provide some evidence that the share of ‘residual users’ has grown significantly in the last two decades. This has been facilitated by generous demand subsidies, enabling private landlords to gain substantial returns in a normally low-revenue market segment. Moreover, in many European countries but also in the US, governments have come to experiment with projects on the provision of market rental accommodation for homeless people (see O’Sullivan and De Decker 2007; Retsinas and Belsky 2008). Arguably, this is not a form of free market renting, since access to those dwellings is defined by social criteria. Nonetheless, this development shows that many tenants, who would have traditionally found accommodation in the social housing sector, now form a new and growing group of potential private renters.

The theoretical statements about social housing do not necessarily align with how it actually works in political, social, and business practices. Housing policies may assign a broader role to social housing than just satisfying housing need. For instance, Sweden, Denmark, and the Netherlands are well known for their social housing sectors which offer housing services to those households that, given their income, would be able to pay for housing services at market levels (Whitehead 2003). Here, social housing is expected to perform as a socially integrating force, preventing the stigmatization of low-income households (Haffner et al. 2009; Whitehead and Scanlon 2007). This links to planning practice in, for instance, the UK, the Netherlands, and Ireland (ibid. 2007; Redmond and Norris 2007), where public housing companies or private housing associations are required to cooperate with private developers and (non-housing) social institutions on building socially mixed neighbourhoods. As a result, new social and market rental accommodation is often provided in identical locations leading to an increased scope for competition.

There are also good grounds to relax the assumption that pecuniary considerations do not play a role in social housing. With the introduction of more market orientated social housing policies the objective functions of social landlords have become much more diverse. As a first step in this development, governments have sought to transfer the ownership and provision to other suppliers than public authorities. In the UK this has involved stock transfers from council suppliers to privately managed Large-Scale Voluntary Transfer associations (Malpass 2010). In the Netherlands municipal stock has been primarily transferred to existing housing associations, while in Sweden significant parts of the municipal stock have been transferred to tenant cooperatives. In business practice the transfer of public stock to private organizations was accompanied by the introduction of private funding schemes and a concurrent reduction of public subsidies in most Western European countries. As a result, social housing organizations are increasingly expected to work along commercial guidelines and generate considerable profits—which, however, have to be fed back into their social activities. It thus seems that they integrate administrative and pecuniary allocation mechanisms.

Based on these rental market ‘realities’ one could argue that from the consumers’ viewpoint, social housing and at least parts of the market rental sector are thus not necessarily ‘worlds apart’. The two rental services might have similar prices, qualities, and locations; or the other way round, providers of market and social housing might have similar customer bases. The contention of this study thus is that the relation between social and market renting has become blurred in some countries—yet, certainly not in all countries—leading to more competitive pressures on both landlord groups.

In the meantime this unclear relation has come to the attention of the European Union’s competition authorities which questioned whether the relation between social and market renting in Sweden and the Netherlands is in accordance with the EU’s competition rules; in other words, whether there is a level playing field for the suppliers of rental housing (Priemus 2008; Lind 2007). Consequently, competition between the two rental tenures is not only possible in business practices but has become a direct subject of political decision-making within countries and on a supranational level.

To conclude, the assumption still holds that competition cannot be based on simple price cuts, since administrative allocation mechanisms dominate in the social sector. Yet, non-price competition between social and market landlords for tenants or a mix of tenants on the basis of, for instance, rent/quality relations, property rights, location, or a combination of those might very well be possible.

### Existing conceptual frameworks

The described development towards a more competitive relationship between the two rental tenures in many countries has been acknowledged by an increasing number of housing researchers (e.g. Boyne and Walker 1999; Murie 2008; Rhodes and Mullins 2009; Hulse et al. 2010). However, there are few conceptual frameworks that guide the analysis of this relationship. Here, the most influential exception has been Kemeny’s (1995) seminal work on unitary and dualist rental markets (see also Kemeny et al. 2005). In brief, the main difference between the two systems is the degree of competition between the profit-oriented and non-profit rental providers. A mature unitary market does not have any regulatory barriers to competition, while in dualist markets a strict separation between the two types of landlords exists.

Some empirical evidence on the effects of a competitive relationship between social and market renting is provided by Atterhoeg and Lind (2004). They test the neoclassical assumption that competition between all sorts of rental providers “would lead to lower prices, reduced costs, more innovation and generally a stronger position for the consumer” (p. 108).

Finally, Haffner et al. (2009; Oxley et al. 2010; Elsinga et al. 2009) conceptualize the meanings and conditions of a competitive relationship between social and market renting through an application of primarily mainstream economic concepts. At the heart of their analysis are the ideas of substitutability between social and market rental services and rivalry between their suppliers. Here, the authors are able to demonstrate the value of the economic concept of competition as a tool of rental housing research in a comparative perspective.

These three conceptual frameworks provide some useful starting points for the analysis of a competitive relationship between social and private renting. However, since they mainly focus on structural and political aspects of competition, they are not able to grasp the behavioural aspects of this relationship. Hence, in order to devise a holistic conceptual framework for analysing inter-tenurial competition on rental housing markets, it seems to be helpful to turn to established competition theories in the economics literature.

## The structure-conduct-performance paradigm

The traditional neoclassical framework for the analysis of competition between providers in fully commercial markets is the structure-conduct-performance (SCP) paradigm. It has been claimed that the SCP can shed light on the competitive conditions of a market in which firms operate, how those conditions affect their behaviour, and what the economic effects of both individual and collective behaviour are (see Motta 2004; Oz 1995). Market structure consists of three aspects (see Clarkson and LeRoy Miller 1983): supply concentration measures the number and market shares of suppliers in a market; product differentiation measures the homogeneity of the products that are being traded; and barriers to entry and exit measure how likely new suppliers enter and exit a market and thus how stable the supply structure in a market is. Firm conduct is defined as the individual firm’s policies towards its product markets and towards the moves made by rival firms. The main questions are how firms set prices—collusively, tacitly, or independently—and which strategies they pursue to discourage entrants (Jacquemin 2000). Performance evaluates whether the firms’ interactions lead to efficient (allocative, productive, and dynamic efficiency are distinguished here), equitable, and consumer-satisfying outcomes in the market (Motta 2004).

The underlying hypothesis of the SCP paradigm is that a stable causal relationship between the three elements exists. The structure of a market is exogenous, so conduct and subsequently performance are structurally determined variables. In neoclassical terms—under the condition that all actors have complete information—a market is perfectly competitive when the number of sellers is high, products are homogeneous, and entry and exit barriers are low. Under these conditions, prices are equal to the marginal costs of production, which leads to an efficient, welfare optimal market outcome (Tirole 1988). In all other market forms, from monopolistic competition to monopolies, suppliers have at least some control over prices, reducing the efficiency of market outcomes (Clarkson and LeRoy Miller 1983).

### Shortcomings of the SCP framework

The problems with this basic assumption are manifold. First, the static, unidirectional relationship between structure and performance, and with it the neoclassical assumption that a large number of sellers will necessarily lead to a more efficient market outcome, was challenged by mainly non-mainstream studies (Jacquemin 2000). In contrast to the SCP, the ‘efficiency-structure hypothesis’—which is associated with the (neo-) Austrian notion of economic competition—assumes that market structure is not an exogenously given factor but depends on the strategic decisions and efficiency of individual firms (Schmalensee 1989). Ultimately, this has led to theoretical advancements through the ‘new industrial organization’ theory, which show that price cuts are not always the ultimate response to more competition. It is rather the case that “with the various types of non-price competition, consumer welfare becomes more multi-dimensional and includes aspects such as the quality of the product and the speed and security of the supply […]” (Jacquemin 2000, p. 6).

Second, the traditional SCP theory presumes that non-rational and non-profit-maximizing behaviour in perfectly competitive markets sharply increases the risk to be driven out of the market. A large corpus of economic literature on the nature of the non-profit firm, however, shows that profit maximization is just one strategy; indeed, non-profit firms are better described as rational optimizers pursuing their non-monetary objectives (Young et al. 2010). This has important implications for their pricing behaviour—after all, they might use other devices for allocating their products than standard fees, such as waiting lists or price discrimination—leading to the impossibility of price competition in mixed markets (Brown and Slivinski 2006).

Finally, the SCP as a mainstream economics model ignores the institutional constraints on what individuals and organizations are able to do in a market (North 1990). Literally every market has an institutional framework that can be described as the rules of the game, limiting the choices that firms can make and structuring the interactions between them. Moreover, neo-institutional economics dismisses the idea of rational behaviour. Building on the insights of behavioural economics the notion prevails that due to unequal information, individuals, even within the same organizations, make diverging choices. Translated into firm behaviour this means that each organization deciphers the market environment differently, i.e. with different perceptions on how competitive their environment is and who their competitors are. This in turn leads to differences in firm behaviour, which can deviate significantly from the market equilibrium (Simon 1986).

These limitations do, however, not mean that the SCP theory cannot be applied to rental housing in order to create a more eclectic framework of competition analysis in rental housing markets. Yet, the previous remarks suggest that several substantial modifications need to be made to the original framework.

## The structure-conduct-performance of rental housing

### Modifications to the traditional framework

The observations on the limitations of the original SCP framework as well as the discussion on competition in rental housing markets in Sect. 2 suggest the following modifications to the original framework (see Table 1). Firstly, since the traditional SCP inadequately addresses explanations of firm behaviour, it follows that when applying the SCP to rental housing it should be utilized as an organizing framework, including a more explicit formalization of strategic firm conduct, thereby considering the idea that competition is also a process of conscious rivalry rather than only a property of market structure. In other words, a clear separation of the meanings of structure, conduct, and performance in rental housing is necessary, which then can set the ground for a systematic empirical testing of the relation between the three elements. Secondly, the SCP of rental housing will give considerable weight to the rules and regulations on rental markets. As said, (neo-) institutional economists have clearly pointed out the importance of regulatory frameworks for firm behaviour. Hence, our framework is in line with the ideas of Gibb and Trebeck (2009) who demonstrate how new rules and regulations can facilitate the analysis of a changing social housing system; however, this idea will be expanded to the whole rental market, considering regulation of the market rental sector. Thirdly, instead of analysing competition in one coherent industry, competition between the providers in two industries in one market is explored. This implies that the different adjustment and allocation mechanisms in the two sectors are part of the framework, meaning that the diverging objective functions of social and private landlords should be made explicit under competitive conduct.

**Table 1:** Comparison of the traditional SCP and the SCP of rental housing markets

	Traditional SCP	SCP of rental housing
General conception	Unidirectional causal relationship Analysis of static competition Supply-side competition framework (antitrust and competition policy)	Utilized as an organizing framework Different market adjustment mechanisms—two industries in one market Supply-side framework; yet, role of the consumer and substitutability are made explicit Existence of submarkets Subject of competition—development sites, funding, customers, mix of tenants?
Market structure	*The firms’ market environment* Supply concentration—number and market share of sellers Barriers to entry and exit—barriers to new competition; stability of the number of sellers Product differentiation—homogeneity of products	*General characteristics of market and social renting* Supply & spatial concentration—number and market share of landlords in each sector, broken down to a neighbourhood level Barriers to entry provision and barriers to access consumption—conditions and rules for landlords and tenants Product differentiation—homogeneity of social and market housing services
Conduct	*Behaviour of the firms with respect to their product market and the actions taken by rivalling suppliers* The way prices are determined independent, tacit, collusive behaviour Decisions on how to gain a competitive advantage (e.g. advertising)	*Behaviour of landlords in both sectors* Business models and objective functions The way prices and quality are set—individually, sector-based, tacit Perceptions of providers in the other sector Reactions of landlords to individual and collective behaviour in the other sector *Behaviour of tenants* Perceptions o the substitutability of social and commercial rental services Moving behaviour of tenants as an exertion of choice
Performance	*Economic and social welfare of the firms’ interactions* Pareto efficiency—allocative, productive, dynamic Equitable outcome Degree of consumer satisfaction	*Economic and social effects of a competitive relation of social and market housing* Landlords economic efficiency, impact of competition on socially desired behaviour of social landlords Tenants—consumers’ satisfaction, equity of outcome Government—policy success or failure

Finally, in contrast to the original SCP, where measuring substitutability is exogenous and primarily relies on the calculation of the cross-price elasticity of demand (Motta 2004), substitutability of market and social housing services is made explicit in this framework. The reason is that in some countries social housing and market renting might be more similar than in other countries. Out of this follows that the strict supply-side view of the traditional SCP is abandoned and consumers and their consumption decisions are given more attention, since tenants’ perceptions and actions are decisive in determining the degree of substitutability between the two rental services.

### Market structure

In line with the traditional SCP in manufacturing or other service industries, market structure in the ‘SCP of rental housing services’ framework deals with an assessment of supply concentration, barriers to entry and product differentiation. There is a strong impetus for analysing supply concentration for the two rental sectors separately—after all, private and social landlords might or might not operate in the same market. The assumption here is that a deconcentrated supply structure is more competitive than for instance a situation in which both industries were characterized by a monopolistic supply structure. It also seems to be meaningful to assess the position of social landlords in the whole rental market: Are they by far the biggest players, or are there private landlords with similar market shares?

In contrast to the original framework, supply concentration does not only deal with the number of firms and their respective market shares, but also with the locations of their housing stocks; i.e. with spatial concentration aspects. The rationale to include these is the fact that (rental) housing is spatially fixed. If social housing were supplied in completely different locations than private renting, the whole rental market would be less competitive than a market environment in which social and private landlords provided housing in the same neighbourhoods.

There are good grounds to follow Arnott’s (1995) judgment that barriers to the provision of market renting are relatively low compared to other industries. Surely, the construction of new rental houses takes time and converting owner-occupied housing into market rental accommodation might be restricted. Yet more importantly, economies of scale and fixed costs are low and incumbent landlords do not savour significant absolute cost advantages. In the context of the relation between market and non-market renting, entry barriers should then be defined as the requirements and preconditions providers have to meet when they seek to offer market or social housing services. When bureaucratic burdens are low for a landlord to operate in both sectors, or to switch from one sector to the other, the market environment is more competitive compared to a rental market where providers are bound to a strict regulation of the types of housing services they may offer. In business practice one entry barrier can be the access of landlords to subsidies in the provision of social housing. Here, the notion of a contestable market (see Baumol 1982) applies when all types of incumbent social landlords as well as other types of landlords are allowed to vie for the provision of new social housing and associated subsidies. Contestability also means that subsidies should be assigned to the bidder with the most efficient and socially desired proposal, regardless of their organizational status. For social landlords, on the other hand, there should be a prohibition on using social housing funds for their engagement in the market rental sector; rather, it should take place along strictly commercial lines, which would imply genuine risks to fail.

A previous contention was that the demand side of the rental market needs to be made explicit in the model, as the competitiveness of a rental market is largely influenced by the question of who might actually consume private and social housing services. Accordingly, the model also includes barriers to access the consumption of social and market renting. Is there free choice for tenants between the two rental services, or do regulatory, public policy, or landlord-induced impediments for tenants to consume either rental service exist? In practice barriers to access the social housing sector might be explicit income limits. From a regulatory viewpoint the application of waiting lists for prospective tenants would be a clear barrier to access the sector. Access barriers for tenants might also be present in the commercial sector, if market landlords set up implicit income barriers and try to exclude lower-income households for whatever reason from the consumption of their housing services. On the other hand, high demand pressures might stifle access to market rental housing. Hence, there is the possibility that exogenous barriers exist, which cannot directly be linked to public policy or landlord behavioural processes.

Product differentiation refers to the idea of substitutability of market and social housing; i.e. how heterogeneous the two services are. This framework follows the approach of Haffner et al. (2009). On the one hand, substitutability considers the differences in social and market rent levels and rent control policies, taking both initial rent setting and rent increases into account. Investigating rents should also include the availability and generosity of housing allowances for lower-income households, since they signify what tenants actually have to pay for their housing consumption. Here, the main question is whether allowances are available for social and private tenants under the exact same conditions or whether the two groups are treated differently. On the other hand, differences in the quality of the rental services need to be taken into account; this comprises both the quality of the dwelling and the quality of the location. If low-standard social dwellings were only offered in deprived neighbourhoods, while high-end market dwellings were mainly located in popular areas, the products would barely be seen as substitutes. Additionally, the similarity of security of tenure for tenants in both sectors is an important aspect of substitutability and thus competitiveness.

By defining product differentiation as bundles of dwelling characteristics, it becomes clear that it is more meaningful to look at the rent/quality relation of social and market rental services than at the rent and quality levels separately. After all, prospective tenants might, for instance, consider a low rent/low quality (high rent/high quality) market housing service and a mid-price/mid-quality social housing service as good substitutes. However, it can be assumed that substitutability generally is a question of the purposes of social and market renting. Where the purpose of social housing is to accommodate a broader clientele than just those in need, the aggregate provision of market services and the aggregate provision of social housing are more likely to cover similar market segments than in countries where social housing does not have this purpose. Furthermore, one should keep in mind that private renting is best understood as a set of submarkets, where different types of landlords cater for different tenants in different locations. It thus follows that, if at all, only parts of the private rental market will have some similarities with the social housing sector, but certainly not all of it. Distinguishing these submarkets thus can help in understanding the scope for competition.

### Conduct in rental housing markets

As described before, since not all landlords are rational profit-maximizers, the strictly neoclassical view of a structurally dependent firm conduct does certainly not reflect rental market realities. Hence, the preceding step to the analysis of the actual behaviour of landlords is to distinguish their different business models and objective functions, and what this implies for competitive behaviour. This covers an analysis of how commercial non-profit landlords operate, set objectives, and react to market signals in comparison to profit-oriented landlords. To guarantee a detailed understanding of conduct there should not only be a distinction between for-profit and non-profit providers, but also between the degrees of commercialization of landlords within the market rental. After all, small-scale individual landlords and corporate investors do have very different business models and goals in the market.

On another point, it might be important to make a distinction between ownership and management. In both sectors there might be an organizational division between owners of the dwelling and those who manage it on a day-to-day basis. An example in the market sector would be a division between a private individual owning the dwelling, while the management of the dwelling is in the hands of a commercial letting agent. In the social sector, on the other hand, it might be the case that the housing stock is owned by a public authority but the management services are outsourced to an independent private organization. Therefore, it should be taken into account whether this division exists and how it might influence strategic decision-making.

Actual competitive firm conduct in the traditional SCP paradigm is circumscribed by the firm’s policy towards its product market and towards the moves made by rivals. When speaking about rivalrous behaviour, business economists claim that the basic condition for a competitive relationship between firms is that they perceive each other as rivals for the patronage of customers and take certain risks to overcome contesters (Baum and Korn 1996). In line with our framework, competitive behaviour would thus mean that social landlords see market landlords as rivals and vie for their customer base (and vice versa; see Table 1). This in turn implies that the existence of a rival social (market) industry might have an impact on strategic behaviour in the market (social) industry. One essential question here is how those competitive pressures are transferred into the decision-making of landlords in either sector: i.e. whether they pursue low rent level strategies; whether they invest in the quality of their dwellings, in order to offer more attractive rent/quality bundles; whether they try to increase market shares through the construction or acquisition of new dwellings; or whether they seek to decrease their business risks by operating in market niches where competitive pressures are comparably low.

In contrast to the traditional SCP, conduct in the SCP of rental housing gives significant weight to the role and behaviour of the tenants. In this line of reasoning market structure sets the general conditions for competition between social and market renting from the households’ viewpoint mainly through the degree of substitutability between the two products. In other words, the substitutability of market and social rental services is a necessary precondition for competition between the two industries. Yet, whether there can be actual competition between the two landlord groups is then strongly influenced by the behaviour of tenants. If the rental market environment is created as one competitive unit, the consequent question would then be whether tenants do actually perceive certain rent/quality bundles of housing services as good substitutes and, most importantly, under which circumstances they are willing to substitute those bundles. This refers to choices tenants make regarding their moving behaviour within the rental market; i.e. whether they move from market rental to social housing accommodation (and vice versa) and why they are willing or not willing to move between the two sectors. Hence, in line with landlord conduct, tenant behaviour in the SCP of rental housing conforms to the notion of behaviour influenced by the actors’ perceptions.

### Performance in rental housing markets

Following the traditional SCP, performance evaluates the effects of competition between the suppliers in a market. More precisely, it is about whether competition between social and market housing providers leads to a more efficient and productive delivery of rented housing services, and whether there is a positive (or negative) effect on rent levels and the services’ quality in the rental market as a whole (see Table 1). This is not to say that a stronger market integration of social and private renting is supposed to be superior; after all, competition with market suppliers might also have a negative impact on the delivery of the public tasks of the social housing industry. For instance, a discrimination of lower-income groups might become a common strategy if social landlords are increasingly dependent on high rent revenues, a development which could ultimately result in the marginalization of lower-income households in the social rental sector. Similarly, one could ask about the effects of a competitive social housing provision on the market rental industry. Does it lead to a decline of market renting, since they are not able to compete with social landlords on equal terms and thus become unprofitable? Or are there any positive effects such as better rent/quality ratios or an expansion in output levels in order to be more competitive than social landlords? Hence, performance is about examining the outcomes of more or less competitive markets and strategies of landlords and moving decisions of tenants on a macro level.

Performance should be assessed from two additional viewpoints. First, from a government’s perspective, performance shows whether the implementation of housing policies that promote competition between the two industries are successful or not. If those policies aim at the creation of separate industries providing housing services for a different set of tenants, then the existence of competitive behaviour would indicate policy failure. Second, performance should be considered from the consumers’ perspective. A competitive rental housing market, in which the two sectors offer housing units that are good substitutes, means that tenants have more choice. If tenants have more choice and if they make use of the choice in their housing consumption decisions, one can assume that the outcome of rental housing allocation satisfies the tenants’ housing preferences. Nonetheless, again it needs to be acknowledged that competition might have negative side-effects. If competition with commercial suppliers induces social housing landlords to focus on more profitable market segments and try to cater for more affluent households in order to secure their own financial stability, this might result in a less equitable rental market, in which social housing suppliers lose sight of their public and social responsibility.

## Applying the conceptual framework to rental housing in the Netherlands: a research agenda

The following discussion considers the applicability of the SCP of rental housing, highlighting some structural and behavioural aspects of competition between the two sectors (see Table 1) in the Netherlands. The aim is not to provide a full-fledged empirical analysis here but rather to set the scene for one. The Netherlands was chosen as a case study for several reasons. On the one hand, there is some evidence that rental housing in the country seems to be relatively competitive (Haffner et al. 2009; Elsinga et al. 2009; Oxley et al. 2010; Kemeny 1995, 2005; Hoekstra 2009). On the other hand, the recent political discussions about the competitive relation between housing associations and private landlords between the EU Commission and the Dutch Government (Priemus and Gruis 2011), as well as the tenure structure in a country with a large social rental sector (31.3 %) and a substantial market rental sector (10.3 %), imply that it is a good choice for a more thorough empirical investigation. Here, this section also raises some questions that could guide a more extensive research approach.

### Market structure

A brief look at supply concentration reveals that 418 housing associations provide social housing in the Netherlands. In the last two decades, intense merger activities between associations have led to a concentration of the sector (Priemus 2003), leading to an average portfolio size of 5,800 dwellings per housing association in 2009. However, the concentration of the social housing sector diverges between urban regions throughout the country. Table 2 shows the market share of the three largest housing associations in the ten largest city regions of the Netherlands. While it is true for all city regions that the social housing supply is strongly concentrated, the concentration ratio of these three associations varies widely: from 44.5 % in the Rotterdam region to 75.7 % in Groningen. Considering the large share of social rental housing in the same regions and assuming a deconcentrated market rental sector (at this stage of the research), it seems that a small number of associations have a dominant position in the country’s city-regional rental markets.

**Table 2:** Concentration of social housing

City region	Market share 3 largest housing associations within social rental sector (%)	Share within rental sector %	
		Social renting	Market renting
Amsterdam	57.5	64.6	35.4
Rotterdam	44.5	75.2	24.8
The Hague	56.4	68.2	31.8
Utrecht	54.1	74.8	25.2
Eindhoven	62.5	86.3	13.7
Tilburg	60.3	80.2	19.8
Groningen	75.7	71.5	28.5
Breda	46.5	77.1	22.9
Nijmegen	72.9	77.2	22.8

*Source* CFV (2011)

Another important aspect of market structure is the question of which tenant groups can consume either rental service. Generally, the Choice-Based Letting allocation scheme in social housing (see Kullberg 2002) stipulates that every citizen older than 18 years can apply for social housing. Yet, there are various barriers to actually access either rental service, where the most profound seems to be the existence of long waiting lists in social housing. The necessary waiting time to get a dwelling differs across households and dwelling types, but can accumulate to more than 10 years in cities like Amsterdam and Utrecht (Elsinga et al. 2009). A further significant barrier to access social housing is the introduction of explicit income limits. Since January 2011, 90 % of all new allocations for social rental dwellings with a rent level of less than € 652 must be allocated to tenants with an income of less than € 33,614 (Priemus and Gruis 2011). The access of middle- and higher-income households to social housing thus has become more restricted. Barriers to access the private sector exist as well, since private landlords often require tenants to fulfil specific income requirements. Furthermore, most tenants face high transaction costs as they have to pay a deposit and a letting agent fee of 1 month’s rent.

Comparing absolute rent levels in the two rental sectors, data from the Dutch Housing Survey for 2009 shows that the average net rent level in the market rental sector (€ 548 per calendar month) is about 50 % higher than mean social housing rents (€ 364 pcm). However, since quality and size are not taken into account here, these relatively large differences could just be a reflection of better and larger dwellings in the private sector. As stated above, under these circumstances substitutability is based on the willingness and ability to pay more for better accommodation.

These three aspects should make it clear that the structural aspects of competition are indeed diverse and complex. Taking into account that the main aim here is to consider the applicability of the framework and not to give a thorough empirical analysis of competitiveness—indeed spatial concentration, barriers to entry provision, quality aspects, and regulation were not mentioned at all—an increasing complexity and interrelation between the aspects of market structure can be assumed and requires a meticulous empirical analysis of structural and political aspects of rental housing. The guiding research question here is formulated as follows:

Research question 1: How competitive is the structure of rental housing markets in the Netherlands with regard to the concentration of supply, the barriers to entry provision, the barriers to access consumption, and the degree of product differentiation between social and market rental services?

### Conduct

Three major groups of rental housing providers exist in the Netherlands. Non-profit housing associations provide mainly social housing (and increasingly market rental accommodation), whereas profit-oriented corporate investors and small-scale individual landlords provide market rental dwellings. It has been pointed out that the business models of the latter groups are very diverse, which is mainly the outcome of the size of their activities (Haffner et al. 2009). Corporate investors (such as pension funds) have very large portfolios, which makes their strategic management choices more observable and accessible. It is thus not surprising that the public discourse on competition between the two rental sectors revolves around the relation between housing associations and corporate investors, while the position of small-scale individual landlords has largely been ignored.

Gruis and Priemus (2008; Priemus and Gruis 2011; Priemus 2008) have extensively discussed the dispute between corporate investors and housing associations about the illegitimacy of state aid for social housing supply. The main argument of corporate investors is that state aid through indirect subsidies (loan guarantees, discounted land prices) would leak into the commercial activities of housing associations and thus create an unlevel playing field in the rental market. According to the authors, at the heart of this dispute is an unclear definition of what social housing is and how it should be distinguished from market rental activities of social landlords. Here, the wider research agenda will apply a qualitative research approach and investigate private and social landlords’ views on the individual sectors, how they relate, and what these views are based on—i.e. how the structure of the rental market affects these views. The notion of rivalry between landlord groups (see Sect. 4) will be used as a guiding theoretical tool:

Research questions, cluster 2: What are the prevailing perceptions of landlords on their rivalrous relationship? How do these perceptions affect landlords’ strategic behaviour in the market? How do structural and political aspects of rental housing, as well as business organizational aspects, affect these perceptions?

With regard to the conduct of tenants and their willingness to substitute social and market rental housing services, Table 3 illustrates that the relative share of moves between social renting and private renting is relatively low compared to the overall number of moves. Nonetheless, within the group of recently moved private renters, a third of them has moved to social housing accommodation. On the other hand, there is also a modest number of social rental households that move to the market rental sector (8 %). The wider research approach seeks to explain this moving behaviour through a quantitative survey among rental households in the Netherlands. Here, the guiding research questions are:

**Table 3:** Moves between housing tenures in the Netherlands (2007–2009)

Current tenure	Previous tenure									
	New households		Owner occupation		Social renting		Market renting		Total	
	*N*	%	*N*	%	*N*	%	*N*	%	*N*	%
Owner occupation	97,459	36	288,263	76	105,937	31	60,084	46	551,743	49
Social renting	126,780	47	63,543	17	204,192	61	**43,617**	**33**	438,132	39
Market renting	45,621	17	26,095	7	**26,790**	**8**	28,040	21	126,546	11
Total	269,860	100	377,901	100	336,919	100	131,741	100	1,116,422	100

*Source* Dutch Housing Survey (Woon) 2009

Bold values indicate the moves from social housing to market renting and vice versa

Research questions, cluster 3: What are tenants’ attitudes to social and market renting? How do these perceptions influence their decision-making on housing consumption? Under which structural and political conditions do tenants actually substitute social and market rental housing services?

**Fig. 1:**
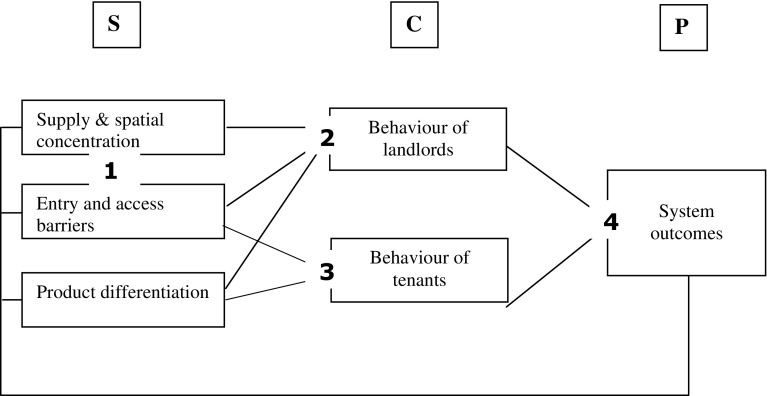
Links between the three elements of the SCP of rented housing. Each line between the *boxes* signifies a possible link. Some of them will be analysed in more detail than others throughout the study. The numbers show the related research question

Figure 1 illustrates the conceptual model and the underlying research questions of the wider research project. Unlike the traditional structure-conduct-performance paradigm, the SCP of rental housing markets does not presuppose a unidirectional causality from structure to performance. Putting conduct in the (empirically) central position of the research approach means that the empirical investigation primarily aims to provide evidence for how structural and political aspects of competition are mediated through actors in the rental market and how their conduct affects the outcomes on the rental market as a whole. Accordingly a fourth cluster of research questions is:

Research questions, cluster 4: What are the effects of the competitive actions and reactions of landlords with regard to tenant satisfaction and the efficiency and equity of rental housing supply? How do these economic and social outcomes affect the structure of rental markets in the long run?

## Conclusions

Rental housing systems in Europe are undergoing significant changes with regard to the roles of social and market renting, their regulatory environments, and provision structures. Those changes have significant consequences for the competitive relation of social and market rented housing. However, conceptual models that allow for a holistic analysis of this phenomenon do not exist. Therefore, on a theoretical basis this paper sought to develop a framework that can be used as an analytical tool to shed light on the economic and political realities of competition between the two rental tenures, whether and how they affect the behaviour of the market actors, and what the system outcomes of a competitive relationship are. It was argued that a rounded industrial organization approach as proposed here, modified by some ideas of institutional economics, might be particularly valuable for policy-makers and regulators, since it is able to put very specific housing political instruments, such as housing allowances or rent regulation, into the broader rental housing market context.

Whether those points regarding the framework are really valid is of course subject to a solid empirical testing of the theory. Hence, by considering the applicability of the framework through a brief discussion of selected competition aspects in the context of rental housing in the Netherlands, the paper set out a wider research agenda and its guiding research questions. It was argued that rental housing in the Netherlands could function as a single case study; however, there might also be a reason to apply the framework in a comparative perspective. Investigating the diverging structural and political environments could help explain the behavioural patterns of competition between social and market renting. The main contention then is that a successful application of the framework, first in a single-country case study and subsequently in a cross-country context, would ultimately yield better understanding of the relation between social and market renting in contemporary rental housing markets.

## Footnotes

1 Similar to Maclennan and More (1997) the terms social and non-market housing are used interchangeably in this paper. There is thus no distinction between the provision of rented housing by public and private non-profit providers when talking about social housing. Furthermore social housing is seen as one part of the rental market, where the other sector is market renting, also labeled private renting. Hereby social and market renting describe allocation mechanisms (see below) and the term rental market has the economic connotation as a system in which goods or services are exchanged between suppliers and customers.

2 In this paper the term private rental housing is defined as housing that is allocated through the market. Rental housing that is provided by private actors to friends, family members or employees and is thus not traded on an open market is not subsumed under the term private rental housing. Private rental housing in this article is to be understood as market rental housing.

## References

[CR1] Arnott R (1995). Time for revisionism on rent control?. Journal of Economic Perspectives.

[CR2] Atterhoeg M, Lind H (2004). How does increased competition on the housing market affect rents? An empirical study concerning Sweden. Housing Studies.

[CR3] Barr N (1998). The economics of the welfare state.

[CR4] Baum J, Korn H (1996). Competitive dynamics of interfirm rivalry. Academy of Management Journal.

[CR5] Baumol WJ (1982). Contestable markets: An uprising in the theory of industry structure. American Economic Review.

[CR6] Boyne GA, Walker RM (1999). Social housing reforms in England and Wales: A public choice evaluation. Urban Studies.

[CR7] Brown E, Slivinski A, Powell W, Steinberg R (2006). Nonprofit organizations and the market. The nonprofit sector: A research handbook.

[CR8] Centraal Fonds Volkshuisversting (CFV). (2011). Regiorapportages (Regional reports for the housing association sector). Accessed under http://www.cfv.nl/publicaties/publicatie/Regiorapportages at 09.10.2011.

[CR9] Clarkson K, LeRoy Miller R (1983). Industrial organization: Theory, evidence, and public policy.

[CR10] Elsinga M, Haffner M, van der Heijden H, Oxley M (2009). How can competition in social rental housing in England and the Netherlands be measured?. European Journal of Housing Policy.

[CR11] Gibb K, Trebeck K (2009). Different roads? Evidence about the changing provision of English social housing. International Journal of Housing Markets and Analysis.

[CR12] Gray P, McAnulty U (2008). The increased role of the private rented sector in catering for low-income groups in Northern Ireland. International Journal of Housing Policy.

[CR13] Gruis V, Priemus H (2008). European competition policy and national housing policies: International implications of the Dutch case. Housing Studies.

[CR14] Haffner M, Boelhouwer P (2006). Housing allowances and economic efficiency. International Journal of Urban and Regional Research.

[CR15] Haffner, M., Hoekstra, J., van der Heijden, H., & Oxley, M. (2009). Bridging the gap between market and social rented housing in six European countries? *Housing and Urban Policy Studies, 33*. Amsterdam: IOS Press.

[CR16] Hoekstra J (2009). Two types of rental system? An exploratory empirical test of Kemeny’s rental system typology. Urban Studies.

[CR55] Hulse K., Jones C. (2010). Tenurial “competition”, tenure dynamics and the private rented sector: An international reappraisal. Journal of European Real Estate Research.

[CR17] Hulse K, Pawson H (2010). Worlds apart? Lower-income households and private renting in Australia and the UK. International Journal of Housing Policy.

[CR18] Jacquemin, A. (2000). *Theories of industrial organisation and competition policy: What are the links?* Working Paper, Forward Studies Unit, European Commission.

[CR19] Kemeny J (1995). From public housing to the social market: Rental policy in comparative perspective.

[CR20] Kemeny J (2005). Non-profit housing influencing, leading and dominating the unitary rental market: Three case studies. Housing Studies.

[CR21] Kemp PA, Kofner S (2010). Contrasting varieties of private renting: England and Germany. International Journal of Housing Policy.

[CR22] Kullberg J (2002). Consumers’ responses to choice-based letting mechanisms. Housing Studies.

[CR23] Lind, H. (2007). The municipal housing companies in Sweden: Current situation and future prospects. Paper presented at the ENHR conference in Rotterdam.

[CR56] Maclennan, D. (1982). *Housing economics: An applied approach*. London: Longman.

[CR24] Maclennan D, More A (1997). The future of social housing: Key economic questions. Housing Studies.

[CR25] Malpass P, Malpass P, Rowland R (2010). The rise (and rise?) of housing associations. Housing, markets and policy.

[CR26] Motta M (2004). Competition policy—theory and practice.

[CR27] Murie, A. (2008). Social housing privatisation in England. In K. Scanlon & C. Whitehead (Eds.), *Social housing in Europe*—*a review of policies and outcomes* (pp. 241–260). London: LSE.

[CR28] North D (1990). Institutions, institutional change and economic performance.

[CR29] O’Sullivan E, De Decker P (2007). Regulating the private rental housing market in Europe. European Journal of Homelessness.

[CR30] O’Sullivan T, Gibb K, O’Sullivan T, Gibb K (2003). Introduction. Housing Economics & Public Policy.

[CR31] Oxley M (2000). The future of social housing: Learning from Europe.

[CR32] Oxley M, Elsinga M, Haffner M, van der Heijden H (2010). Competition and social rented housing. Housing, Theory and Society.

[CR33] Oz S (1995). Industrial organization—theory and applications.

[CR34] Pawson H (2006). Restructuring England’s social housing sector since 1989: Undermining or underpinning the fundamentals of public housing?. Housing Studies.

[CR35] Priemus H (2003). Dutch housing associations: Current developments and debates. Housing Studies.

[CR36] Priemus H (2008). Real estate investors and housing associations: A level playing field? The Dutch case. European Journal of Housing Policy.

[CR37] Priemus H, Gruis V (2011). Social housing and illegal state aid: The agreement between European Commission and Dutch Government. European Journal of Housing Policy.

[CR38] Quigley J, O’Sullivan T, Gibb K (2003). Transactions costs and housing markets. Housing economics and public policy.

[CR39] Redmond, D., & Norris, M. (2007). Social housing in the Republic of Ireland. In C. Whitehead & K. Scanlon (Eds.), *Social housing in Europe I* (pp. 118–129). London: LSE.

[CR40] Retsinas N, Belsky B (2008). Revisiting rental housing—policies, programs, and priorities.

[CR41] Rhodes M, Mullins D (2009). Market concepts, coordination mechanisms and new actors in social housing. International Journal of Housing Policy.

[CR42] Rugg, J., & Rhodes, D. (2008). *The private rented sector: Its contribution and potential*. York Centre for Housing Policy, University of York.

[CR43] Scanlon K, Whitehead C (2008). Social housing in Europe II—a review of policies and outcomes.

[CR44] Schmalensee R, Schmalensee R, Willig R (1989). Inter-industry studies of structure and performance. Handbook of industrial organization, volume II.

[CR45] Simon H. (1986). Rationality in psychology and economics. Journal of Business.

[CR46] Tirole J (1988). The theory of industrial organization.

[CR47] Turner, B. (2007). Social housing in Sweden. In C. Whitehead & K. Scanlon (Eds.), *Social housing in Europe I* (pp. 148–164). London: LSE.

[CR48] Walker R (2000). The changing management of social housing: The impact of externalisation and managerialisation. Housing Studies.

[CR49] Whitehead C, O’Sullivan T, Gibb K (2003). The economics of social housing. Housing economics and public policy.

[CR50] Whitehead C, Scanlon K (2007). Social housing in Europe I.

[CR51] Young D, Jung T, Aranson R (2010). Mission-market tensions and nonprofit pricing. The American Review of Public Administration.

